# Duration of birth depression and neurodevelopmental outcomes after whole-body hypothermia for hypoxic ischemic encephalopathy in India, Sri Lanka and Bangladesh – an exploratory analysis of the HELIX trial

**DOI:** 10.1016/j.lansea.2023.100284

**Published:** 2023-10-04

**Authors:** Constance Burgod, Munirah Mazlan, Stuti Pant, Vaisakh Krishnan, Reema Garegrat, Paolo Montaldo, Pallavi Muraleedharan, Prathik Bandiya, Chinnathambi N. Kamalaratnam, Rema Chandramohan, Swati Manerkar, Ismat Jahan, Sadeka C. Moni, Mohammod Shahidullah, Ranmali Rodrigo, Samanmali Sumanasena, Radhika Sujatha, Babu Peter Sathyanathan, Anagha R. Joshi, Ronit R. Pressler, Paul Bassett, Seetha Shankaran, Sudhin Thayyil

**Affiliations:** a Centre for Perinatal Neuroscience, Imperial College, London, United Kingdom; b Neonatal Unit, Indira Gandhi Institute of Child Health, Bangalore, India; c Neonatal Unit and Radiology, Madras Medical College, Chennai, India; d Neonatal Unit and Radiology, Lokmanya Tilak Municipal Medical College, Mumbai, India; e Neonatal Unit, Bangabandhu Sheikh Mujib Medical University, Bangladesh; f Neonatal Unit, University of Kelaniya, Sri Lanka; g Neonatal Unit, Sree Avittom Thirunal Hospital and Government Medical College, Thiruvananthapuram, India; h Department of Neurophysiology, Great Ormond Street Hospital, United Kingdom; i Statsconsultancy Ltd, Amersham, United Kingdom; j Department of Neonatal-Perinatal Medicine, Wayne State University, Detroit, MI, USA; k University of Texas at Austin, Dell Children's Hospital, Austin, USA

**Keywords:** Hypoxic ischaemic encephalopathy, Newborn, Magnetic resonance

## Abstract

**Background:**

Effect of duration of birth depression on neurodevelopmental outcomes in low- and middle-income countries (LMICs) is not known. We examined the association of birth depression with brain injury, neurodevelopmental outcomes, and hypothermia after hypoxic ischemic encephalopathy (HIE) in south Asia.

**Methods:**

We compared cerebral magnetic resonance (MR) at 2 weeks, and adverse outcomes (death or moderate or severe disability) at 18 months in 408 babies with moderate or severe HIE who had long birth depression (positive pressure ventilation (PPV) >10 min or Apgar score<6 at 10 min or cord pH < 7.0) and short birth depression (PPV for 5–10 min or Apgar score<6 at 5 min, but ≥6 at 10 min).

**Findings:**

Long depression group (n = 201) had more severe HIE (32.8% versus 6.8%), mortality (47.5% versus 26.4%), death or disability at 18 months (62.2% versus 35.4%) (all p < 0.001), MR injury (Odds ratio; 95% CI) to basal ganglia (2.4 (1.3, 4.1); p = 0.003), posterior limb of internal capsule (2.3 (1.3, 4.3); p < 0.001) and white matter (1.7 (1.1, 2.7); p = 0.021), and lower thalamic N-acetylaspartate levels (7.69 ± 1.84 versus 8.29 ± 1.60); p = 0.031) than short depression group (n = 207). Three babies had no heartbeat at 5 min, of which 1 died and 2 survived with severe disability. No significant interaction between the duration of birth depression and whole-body hypothermia was seen for any of the MR biomarker or clinical outcomes.

**Interpretation:**

Long birth depression was associated with more brain injury and adverse outcomes than short depression. Effect of hypothermia was not modified by duration of birth depression.

**Funding:**

10.13039/501100000272National Institute for Health Research.

**Research in context**

**Evidence before this study**

We examined the studies included in a recent systematic review on outcomes after hypoxic ischemic encephalopathy from low- and middle-income countries (LMICs)^1^ and updated the search on 1 Aug 2023 to identify any additional studies. Total of 5 studies reported neurodevelopmental outcomes after hypoxic ischemic encephalopathy at 18 months or more. None of the studies reported the effects of long or short birth depression on clinical outcomes, brain injury or response to whole-body hypothermia.

**Added value of this study**

We found that the babies with long birth depression more often had severe encephalopathy, higher mortality, and more death or moderate or severe disability at 18 months than those with short depression. Amongst babies who survived to have magnetic resonance (MR) imaging at 1–2 weeks, brain injury on conventional MR imaging scores and spectroscopy was worse in those who had long birth depression than in those with short birth depression. All babies without a heart rate at 5 min of resuscitation after birth either died or had severe disability. Whole-body hypothermia did not improve brain injury or clinical outcome irrespective of the duration of birth depression.

**Implications of all the available evidence**

Both long and short birth depression are associated with adverse outcomes in LMICs although the adverse outcomes are much higher amongst babies who had long birth depression. Whole-body hypothermia did not improve brain injury, or clinical outcomes irrespective of the duration of birth depression. Poor outcomes of babies without a heart rate at 5 min may inform clinical decision making during neonatal resuscitation in LMICs. Careful experimental research into mechanisms and clinical heterogeneity may be required to develop future preventive and therapeutic strategies for hypoxic ischemic encephalopathy in LMICs.

## Introduction

Hypoxic ischemic encephalopathy (HIE) is a serious neurological condition arising from substantial intra-uterine hypoxia-ischemia to the fetal brain immediately prior to the delivery leading to birth depression, and accounts for one million deaths every year.^2^ HIE occurs in around 1 to 2 per 1000 livebirths in high income countries (HICs).^3^ In most cases, HIE is unpredictable and is related to a sudden and unexpected intra-partum hypoxic event after an otherwise uncomplicated pregnancy.

Therapeutic hypothermia is the standard treatment for babies with moderate or severe HIE based on evidence from clinical trials conducted in HICs in the past two decades.^4^, ^5^, ^6^, ^7^ In contrast, a large multi-country clinical trial from India, Sri Lanka and Bangladesh (Hypothermia for encephalopathy in low and middle-income countries trial – HELIX trial)^8^ found no evidence that whole-body cooling in these settings reduced death or disability at 18 months in babies with HIE (risk ratio 1.06; 95% CI 0.87–1.30; p = 0.55), but increased mortality (risk ratio 1.35 (1.04–1.76); p = 0.022). We hypothesised that babies who had long birth depression would have worse clinical outcomes and poorer response to hypothermic neuroprotection than those who had short birth depression.

In this exploratory analysis of the HELIX trial, we examined the association of long and short birth depression with neonatal brain injury and neurodevelopmental outcomes after moderate or severe HIE. We also examined if the response to whole-body hypothermia differed in babies who had long compared to short birth depression.

## Methods

### Study design and participants

The HELIX study^8^ was an open-label, multi-country, randomised controlled trial that recruited a total of 408 term and near-term babies presenting with moderate or severe encephalopathy and randomised them to whole-body hypothermia (33.5 °C for 72 h) or control group (36.5 °C), within 6 h of birth. The study took place in seven tertiary neonatal intensive care units in India, Sri Lanka, and Bangladesh from August 2015 to February 2019. Babies born at or after 36 weeks with a minimum birth weight of 1.8 kg were recruited to the study if parental consent was obtained and if they required continued resuscitation at 5 min of age or had an 5-min Apgar score less than 6 (or both) or lack of cry by 5 min for those born at home, and if a structured neurological examination performed 1–6 h after birth by a certified examiner indicated evidence of moderate or severe HIE. We excluded infants who had no heart rate at 10 min of age despite adequate resuscitation, those with major life-threatening congenital malformations, or if the parents were unable to attend follow-up assessments.

All participating sites had facilities for assisted ventilation and continuous cardiovascular monitoring and were designated tertiary care regional neonatal intensive care units.

In this subgroup exploratory analysis we defined, long birth depression as: requiring positive pressure ventilation (PPV) >10 min or Apgar score <6 at 10 min or an umbilical cord blood pH < 7.0 and short birth depression as: requiring PPV for 5–10 min or Apgar score <6 at 5 min, but ≥6 at 10 min.

### Procedures

All babies had standardised clinical neurological assessment using modified Sarnat staging for encephalopathy (none, mild, moderate, or severe) within 6 h of age at days 1, 2, 3, 4, 7 and at discharge, by a trained and certified examiner. Short term clinical outcomes included death before hospital discharge, major intracranial haemorrhage on cranial ultrasonography, gastric bleeds, persistent hypotension, pulmonary haemorrhage, persistent pulmonary hypertension, long blood coagulation requiring blood products, culture proven early onset sepsis, necrotising enterocolitis, cardiac arrythmia, severe thrombocytopenia, persistent metabolic acidosis, pneumonia, renal failure, subcutaneous fat necrosis, an abnormal neurological examination at discharge and duration of hospital stay.

**Fig. 1:**
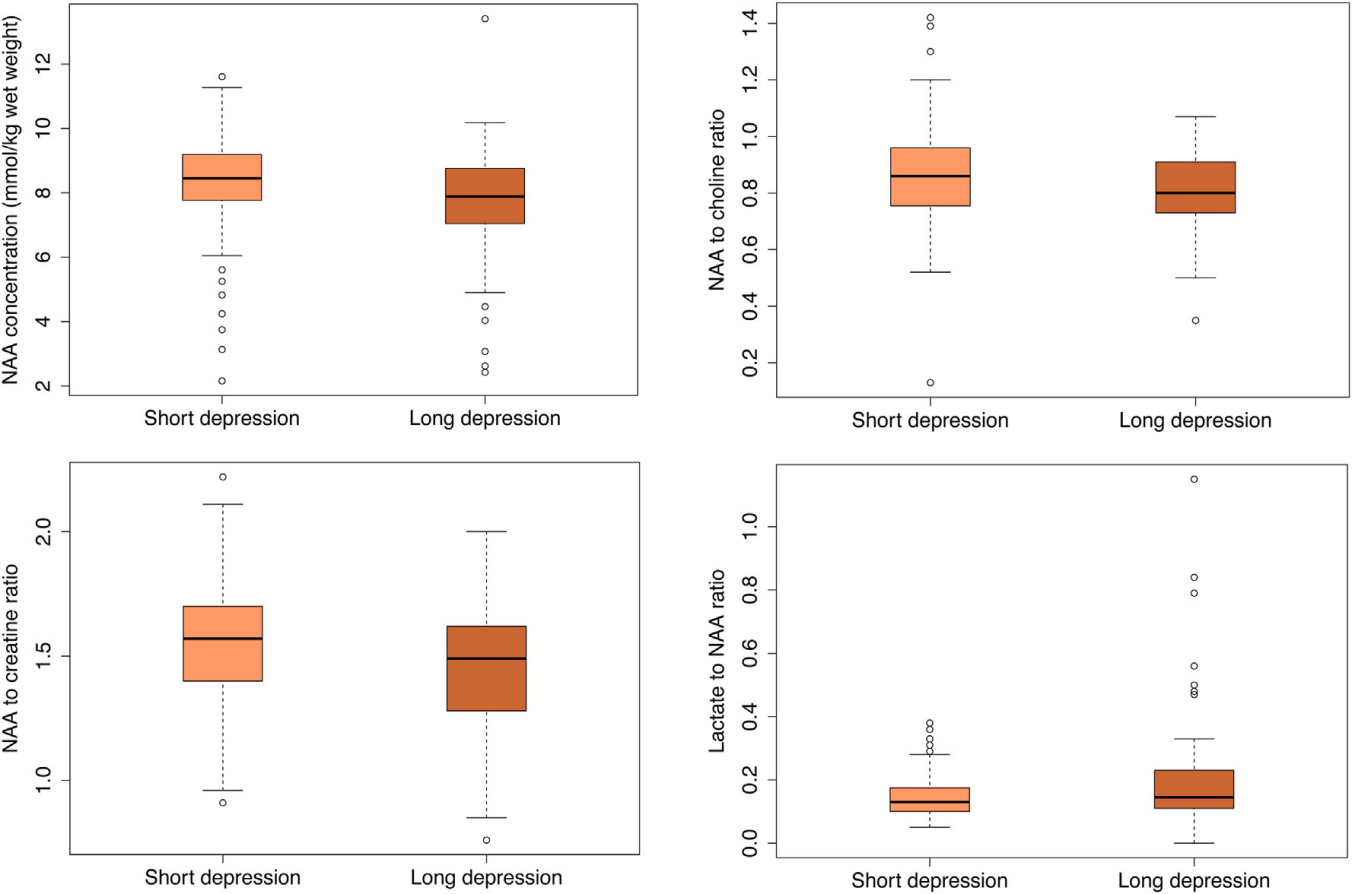
**Proton MR spectroscopy thalamic N-acetyl aspartate (NAA) absolute levels (mmol/kg/wet weight) (top left), NAA/Choline peak area metabolite ratio (top right), NAA/Creatine peak area metabolite ratio (bottom left), lactate/NAA peak area metabolite ratio (bottom right) in long birth depression and short birth depression groups**.

Magnetic Resonance (MR) Imaging was performed using a 3 T scanner on all babies 1–2 weeks after birth unless they had died before the scan. The MR protocol (acquisition time) comprised T1-weighted and T2-weighted MR (15 min) in axial and sagittal planes, diffusion tensor imaging (DTI; 7 min), proton MR spectroscopy metabolite peak area ratios (7 min) and metabolite absolute concentrations (13 min) (Fig. 1).^9^ MR spectroscopy was acquired in a single 15 × 15 × 15 mm^3^ voxel centred on the left thalamus. Predefined quality checks were performed on pseudonymised raw MR data, masked to the allocation and clinical outcome, to exclude poor-quality data whilst avoiding any selection bias. The MR spectroscopy and DTI data were analysed using Linear Combination Model (version 6.3) and Functional MRI of the Brain Software Library (version 6.0), respectively. All MR images were centrally reported using a previously validated scoring system, masked to clinical data.^9^ Basal ganglia and thalamic score: 0 = normal, 1 = mild (focal abnormal signal intensity), 2 = moderate (multifocal abnormal signal intensity), 3 = severe (widespread abnormal signal intensity). White matter score: 0 = normal, 1 = mild (exaggerated long T1 and long T2 in periventricular white matter only), 2 = moderate (long T1 and long T2 extending out to subcortical white matter and/or focal punctate lesions or focal area of infarction), 3 = severe widespread abnormalities including overt infarction, haemorrhage, and long T1 and long T2. Cortical involvement was scored as the presence of abnormal signal intensity, usually decreased T1 or cortical highlighting. 0 = normal, 1 = mild (1–2 sites involved), 2 = moderate (3 sites involved), 3 = severe (more than 3 sites involved).^8^

Neurodevelopmental outcome assessment was performed inappropriate local languages by trained and certified neurodevelopmental paediatricians masked to the allocation group. The primary outcome of the HELIX trial was a composite endpoint of death, or moderate or severe disability assessed between 18 and 22 months by the Bayley Scales of Infant and Toddler Development, third edition (Bayley-III).^10^ Severe disability was defined as any of the following: a cognitive composite score of less than 70, a gross motor function classification system level 3–5,^11^ a profound hearing impairment requiring hearing aids or a cochlear implant, or blindness. Moderate disability was defined as a cognitive compositive score of 70–84 and one or more of the following: a gross motor function classification system level 2, a hearing impairment with no amplification, or a persistent seizure disorder.

### Statistical analysis

The baseline clinical characteristics and outcomes of the long depression group and short depression group were compared using the unpaired t-test (normal distribution) or Mann–Whitney U test (skewed distribution) for continuous variable, and the Chi-square test or Fisher's exact test (if outcome per group occurred in less than 5 cases) for categorical variables.

MR biomarkers within long depression and short depression group are expressed as odds ratios and mean difference with corresponding 95% confidence intervals. Binary outcomes were compared between groups using the Chi-square test, with group differences expressed as proportional differences with 95% confidence intervals. Ordinal logistic regression was used for the analysis of ordinal outcomes whilst continuous outcomes were compared between groups using the unpaired t-test. The distribution of the birth depression groups (long and short) within the whole-body hypothermia and control groups were also examined.

Finally, we explored how the impact of whole-body hypothermia varied based on the birth depression subgroup of the baby. The first analysis examined how the distribution of the birth depression subgroups varied in two study groups [whole-body hypothermia or control]. This association was examined using the Chi-square test. We then examined whether or not the effect of the study intervention [whole-body hypothermia] varied in the two birth depression groups [long or short depression]. This was examined using a regression approach. The study (randomisation) group and birth depression group were included as fixed factors, along with the interaction between the two factors. A significant interaction would imply that effects of the study group varied for the two birth depression groups, while p values > 0.05 suggests no evidence of an interaction.

Binary outcomes were analysed using logistic regression, whilst ordinal outcomes were analysed using ordinal logistic regression. Linear regression was used for continuous outcomes, and where necessary, continuous outcomes with a positively skewed distribution were given to a log transformation before analysis. Data were analysed using Stata software, version 17.0 (StataCorp LLC).

### Role of the funding source

The funders of the study had no role in study design, data collection, data analysis, data interpretation, or writing the report.

## Results

A total of 408 babies were recruited to the HELIX trial, of which 201 (49.3%) had long birth depression and 207 (50.7%) had short birth depression. Mean (SD) birth weight, gestational age and head circumference at randomisation were similar in both groups (Table 1).

**Table 1:** Baseline clinical characteristics of babies in the long and short birth depression groups.

	Long birth depression group (n = 201)	Short birth depression group (n = 207)	Mean/% difference^b^	p value
Maternal age, year	25.0 ± 4.7	23.7 ± 4.4	1.3 (0.4, −2.2)	0.006
Booked pregnancies	194/201 (96.5%)	192/204 (94.1%)	2.4 (−1.7, 6.5)	0.254
Primigravida	116/200 (58.0%)	119/207 (57.5%)	0.5 (−9.1, 10.1)	0.917
Diabetes	2/201 (1.0%)	0/207 (0.0%)	1.0 (−0.01, 0.03)	0.242
Maternal pyrexia	7/191 (3.7%)	1/197 (0.5%)	3.2 (0.3, 6.0)	0.029
Rupture of membranes >24 h	1/189 (0.5%)	3/195 (1.5%)	−1.0 (−0.03, 0.01)	0.606
Meconium-stained liquor	69/193 (35.8%)	41/199 (20.6%)	15.1 (6.4, 23)	0.001
Reduced fetal movements	17/165 (10.3%)	7/170 (4.1%)	6.2 (0.7, 11.7)	0.028
Funisitis	27/179 (15.1%)	39/187 (20.8%)	−5.7 (−0.12, 0.02)	0.151
Perinatal sentinel events	34/201 (16.9%)	9/207 (4.3%)	12.6 (0.06, 0.18)	<0.001
Instrumental delivery	29/201 (14.4%)	11/205 (5.4%)	9.1 (3.3, 14.8)	0.002
Caesarean delivery	57/201 (28.4%)	26/205 (12.7%)	15.6 (8.0, 23.4)	<0.001
**Infant size and condition**				
Birth weight, grams	2863 ± 479	2919 ± 429	−56 (−144, 32)	0.216
Gestational age, weeks	38.9 ± 1.4	38.9 ± 1.2	0.0 (−0.3, 0.2)	0.856
Head circumference, cm	34.3 ± 1.6	34.2 ± 1.4	0.1 (−0.2, 0.4)	0.447
Age at admission to the NICU, minutes	114.4 ± 97.7	178.7 ± 85.3	−64.3 (−82.1, −46.4)	<0.001
Cord pH^b^	6.9 ± 0.2 (n = 43)	7.1 ± 0.1 (n = 3)	−0.2 (−0.4, 0.1)	0.261
Intubation at birth	178/201 (88.6%)	0/200 (0.0%)	88.6 (0.83, 0.92)	<0.001
Cardiac massage	35/201 (17.4%)	8/200 (4.0%)	13.4 (0.08, 0.19)	<0.001
Drugs during resuscitation	43/201 (21.4%)	6/200 (3.0%)	18.4 (12.2, 0.24)	<0.001
Males	129/201 (64.2%)	138/207 (66.7%)	−2.5 (−11.7, 6.7)	0.604
Induced hypothermia	100/201 (49.8%)	102/207 (49.3%)	0.5 (−0.09, 0.10)	1.000
Severe encephalopathy	66/201 (32.8%)	14/207 (6.8%)	26.1 (18.7, 33.4)	<0.001
Seizures at randomisation	135/201 (67.2%)	164/207 (79.2%)	−12.1 (−20.6, −3.5)	0.007
Age of seizure onset^a^	2 [1, 5]	2 [1, 4]	0 (0.0, 0.5)	0.576

a Figures reported are median [inter-quartile range] and median difference (95% confidence interval).

b Data are based on 43 babies in long birth depression group and 3 babies in short birth depression group.

**Fig. 2:**
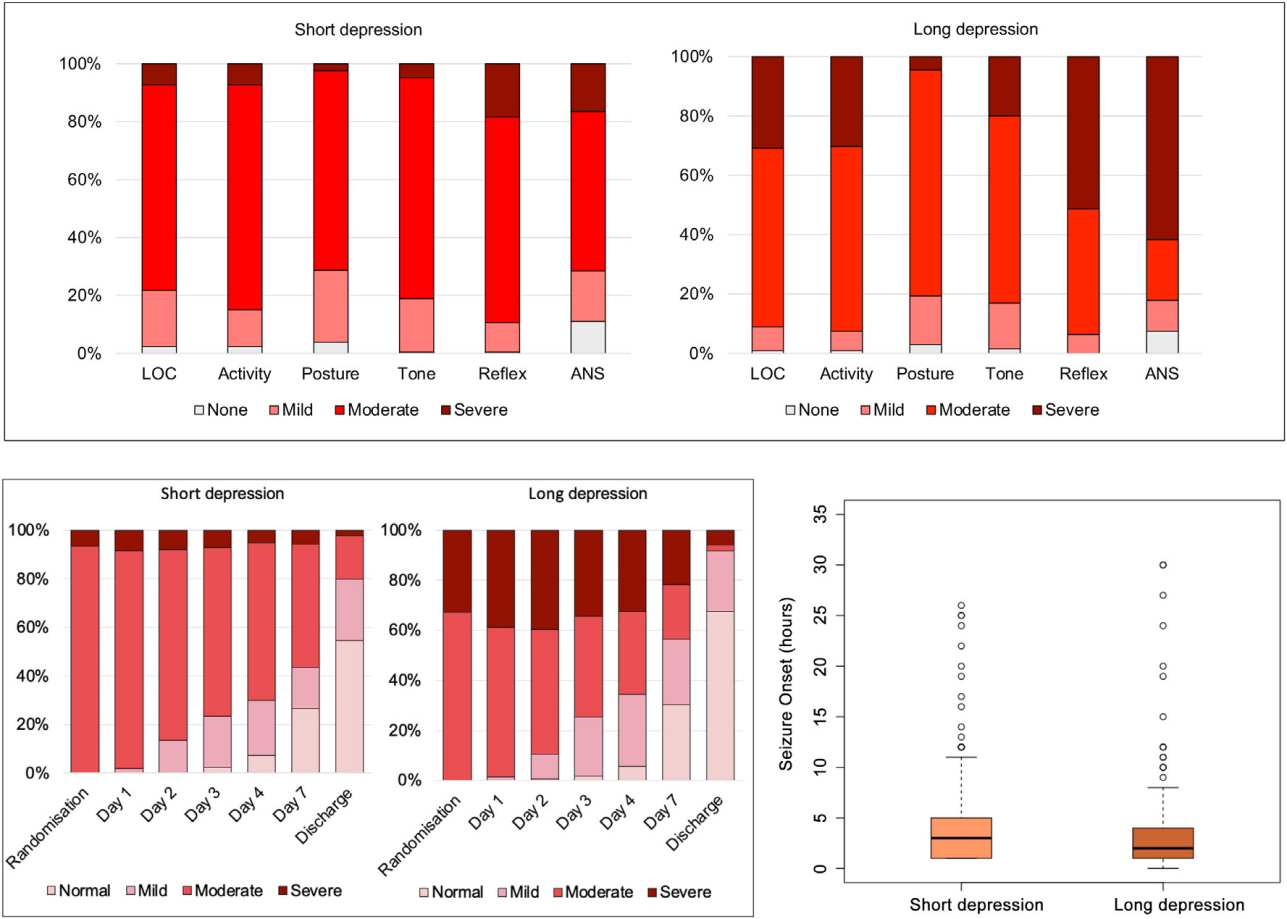
**Six subcategories of the modified Sarnat staging at randomisation indicating the level of abnormality within each subcategory in long birth depression and short birth depression groups is shown in the top panel. The term LOC indicates level of consciousness and ANS indicates autonomic nervous system. Infants only qualified for cooling if they had at least 3 of the 6 subcategories classified as moderate or severe at randomisation. Progression of encephalopathy stage during neonatal hospitalisation in long birth depression and short birth depression groups is shown in bottom left panel, and the median (IQR) of age of seizure onset in the bottom right panel (five outliers with seizure onset above 36 h were excluded from the plot)**.

The long birth depression group had more meconium-stained liquor, perinatal sentinel events, instrumental and caesarean deliveries than the short birth depression group. Babies in the long birth depression group were admitted to the neonatal intensive care unit earlier than the short birth depression group. Intubation at birth, cardiac massage during resuscitation, and the use of drugs during resuscitation were higher in the long birth depression group compared to the short birth depression group. The long birth depression group had more babies with severe encephalopathy at randomisation than the short birth depression group (Table 1; Fig. 2). More clinical seizures at randomisation were reported in the short birth depression group than in the long birth depression group, although median age of seizure onset did not differ between groups (Table 1; Fig. 2).

MRI was available from 116 (57.7%) out of 201 babies from the long birth depression group (51 hypothermia; 65 controls); 75 (37.3%) died, and 5 (2.5%) were discharged against medical advice before the scan could be performed. Data could not be retrieved for the remaining 5 (2.5%). Median (IQR) age at MRI was 13 (10–16) days in the long birth depression group.

In the short birth depression group, MRI was available from 151 (72.9%) out of 207 babies (71 hypothermia; 80 controls); 39 (18.8%) died, and 3 (1.4%) were discharged against medical advice before the scan could be performed. Data could not be retrieved for 12 (5.8%) and consent for MRI was not obtained from the remaining 2 (1.0%) babies. Median (IQR) age at MRI was 14.5 (11–22) days in the short birth depression group. Clinical characteristics and outcomes of the babies with and without MRI are given in the supplementary table.

Babies in the long birth depression group had more injury to basal ganglia, posterior limb of internal capsule, and white matter than those in the short birth depression group on conventional T1-and T2-weighted images. Cortical injury scores were not found to be significantly different between the two groups (Table 2).

**Table 2:** Magnetic resonance imaging, spectroscopy, and clinical outcomes in the neonatal period.

Conventional MRI	Score	Long birth depression group (n = 116)	Short birth depression group (n = 151)	Odds ratio (95% CI)^c^	p value
		n (%)	n (%)		
Basal ganglia and thalamic injury	0	79 (68.1%)	125 (82.8%)	2.36 (1.34, 4.18)	0.003
	1	8 (6.9%)	9 (6.0%)		
	2	14 (12.1%)	11 (7.3%)		
	3	15 (12.9%)	6 (4.0%)		
Posterior limb of internal capsule	Normal	84 (72.4%)	129 (85.4%)	2.33 (1.27, 4.27)	0.0006
	Equivocal	4 (3.4%)	7 (4.6%)		
	Abnormal	28 (24.1%)	15 (9.9%)		
White matter injury	0	20 (17.2%)	35 (23.2%)	1.71 (1.09, 2.69)	0.021
	1	20 (17.2%)	38 (25.2%)		
	2	59 (50.9%)	65 (43.0%)		
	3	17 (14.7%)	13 (8.6%)		
Cortical injury	0	80 (69.0%)	110 (72.8%)	1.36 (0.81, 2.30)	0.249
	1	15 (12.9%)	30 (19.9%)		
	2	8 (6.9%)	4 (2.6%)		
	3	13 (11.2%)	7 (4.6%)		

MR spectroscopy	Mean ± SD	Mean ± SD	Mean difference (95% CI)	p value
[NAA] mmol/kg/ww	7.69 ± 1.84	8.29 ± 1.60	−0.60 (−1.14, −0.06)	0.031
NAA/Choline	0.81 ± 0.15	0.86 ± 0.18	−0.06 (−0.11, −0.01)	0.031
NAA/Creatine	1.46 ± 0.29	1.55 ± 0.25	−0.09 (−0.18, −0.01)	0.026
	**Median (IQR)**	**Median (IQR)**	**Median difference (95% CI)**	

Lactate/NAA^a^	0.15 [0.11, 0.23]	0.13 [0.10, 0.18]	0.00 (0.00, 0.04)	0.044

Outcomes during neonatal hospitalisation	Long birth depression group (n = 201)	Short birth depression group (n = 207)	Percentage difference (95% CI)^b^	p value
Death during hospitalisation	81/201 (40.3%)	40/207 (19.3%)	21.0 (12.3, 29.6)	<0.001
Gastric bleeds	52/201 (25.9%)	44/207 (21.3%)	4.6 (−3.6, 12.8)	0.295
Persistent hypotension	43/201 (21.4%)	27/207 (13.0%)	8.3 (1.1, 15.6)	0.026
Pulmonary haemorrhage	43/201 (21.4%)	27/207 (13.0%)	8.3 (1.1, 15.6)	0.026
Persistent pulmonary hypertension	29/201 (14.4%)	11/207 (5.3%)	9.1 (3.3, 14.8)	0.002
Severe thrombocytopenia	26/201 (12.9%)	22/207 (10.6%)	2.3 (−3.9,8.6)	0.539
Persistent metabolic acidosis	38/201 (18.9%)	32/207 (15.5%)	3.4 (−3.9, 10.8)	0.362
**Outcomes at 18 months**				
Death	94/198 (47.5%)	53/201 (26.4%)	21.1 (11.9, 30.4)	<0.001
Amongst moderate encephalopathy	44/132 (33.3%)	42/188 (22.3%)	−11.0 (−21.0, −1.0)	0.03
Amongst severe encephalopathy	50/66 (75.7%)	11/13 (84.6%)	8.9 (−13.3, 31.0)	0.49
Death or moderate or severe disability	122/196 (62.2%)	70/198 (35.4%)	26.8 (17.4, 36.4)	<0.001
Amongst moderate encephalopathy	61/130 (46.9%)	59/185 (31.9%)	−15.0 (−25.9, −4.1)	0.007
Amongst severe encephalopathy	61/66 (92.4%)	11/13 (84.6%)	−7.8 (−28.4, 12.8)	0.37
Cerebral palsy	27/102 (26.5%)	13/145 (9.0%)	17.5 (7.8, 27.2)	<0.001
Microcephaly	40/102 (39.2%)	30/143 (21.0%)	18.2 (6.7, 29.8)	0.002
Wasting	51/101 (50.5%)	38 (26.6%)	23.9 (11.8, 36.1)	<0.001
Stunting	68/102 (66.7%)	59/142 (41.6%)	25.1 (12.9, 37.3)	<0.001

Basal ganglia and thalamic score: 0 = normal, 1 = mild (focal abnormal signal intensity), 2 = moderate (multifocal abnormal signal intensity), 3 = severe (widespread abnormal signal intensity).

White matter score: 0 = normal, 1 = mild (exaggerated long T1 and long T2 in periventricular white matter only), 2 = moderate (long T1 and long T2 extending out to subcortical white matter and/or focal punctate lesions or focal area of infarction), 3 = severe widespread abnormalities including overt infarction, haemorrhage, and long T1 and long T2.

Cortical involvement was scored as the presence of abnormal signal intensity, usually decreased T1 or cortical highlighting. 0 = normal, 1 = mild (1–2 sites involved), 2 = moderate (3 sites involved), 3 = severe (more than 3 sites involved).

The term microcephaly indicates a head circumference of smaller than 2 SDs less than the mean for their age, based on WHO child growth charts in 2009.^12^ The term wasting indicates a weight less than the fifth centile for their age and stunting indicates a height (length) less than the fifth centile for their age, based on WHO child growth charts in 2009.^12^

a Figures reported are median [inter-quartile range] and median difference (95% confidence interval).

b Percentage difference calculated as long birth depression group—short birth depression group.

c Odds Ratios calculated as the odds of being in the next highest outcome category for long birth depression group relative to the odds of being in the next outcome category for short birth depression group were reported for conventional MR imaging.

The mean (SD) N-acetylaspartate [NAA] in mmol/kg/ww, NAA/Choline and NAA/Creatine were lower in the long birth depression group compared to the short birth depression group. Median (IQR) Lactate/NAA was higher in the long birth depression group compared to the short birth depression group (Table 2; Fig. 1).

More babies in the long birth depression group died during hospitalisation, had persistent hypotension, pulmonary haemorrhage, and persistent pulmonary hypertension than short birth depression group (Table 2). At 18 months, more babies in the long birth depression group died and had the composite endpoint of death or moderate or severe disability. Cerebral palsy, microcephaly, and growth retardation were also higher in the long birth depression group than the short birth depression group (Table 2). In two babies (both in the long birth depression group), first heartbeat was present only after 5 min of resuscitation after birth (1 died and 1 had severe disability) and another baby had a heartbeat only after 10 min of resuscitation after birth (survived with severe disability).

Proportion of babies receiving whole body hypothermia or usual intensive care in the long birth depression group and the short birth depression group were not different. No evidence of significant interaction of the duration of birth depression group (long birth depression or short birth depression) on the study group (control or whole-body hypothermia) was seen for any of the MR biomarker or clinical outcomes (Table 3). Hence, the effects of whole-body hypothermia in the long and short birth depression groups were not quantified separately.

**Table 3:** Interactions between study (randomisation to hypothermia and control) group and birth depression (long or short) group.

Outcome group	Outcome variable	Birth depression group x randomisation group interaction p value
Clinical Outcomes	Death during hospitalization	0.25
	Gastric bleeds	0.23
	Persistent hypotension	0.40
	Pulmonary haemorrhage	0.70
	Persistent pulmonary hypertension	0.31
	Severe thrombocytopenia	0.57
	Persistent metabolic acidosis	0.61
	Death up until 18 months	0.45
	Death or moderate or severe disability	0.71
Conventional MRI	Basal ganglia and thalamic injury	0.95
	Posterior limb of internal capsule	0.87
	White matter injury	0.48
	Cortical injury	0.63
MR Spectroscopy	[NAA] mmol/kg/ww	0.30
	NAA/Choline	0.55
	NAA/Creatine	0.32
	Lactate/NAA	0.53

## Discussion

In this subgroup exploratory analysis of the HELIX trial,^8^ we report three important observations. Firstly, there were differences between the short and long birth depression groups. The long birth depression group had more babies with severe encephalopathy (32.8% versus 6.8%; p ≤ 0.001), higher mortality (47.5% versus 26.4%; p ≤ 0.001) and higher death or disability at 18 months (62.2% versus 35.4%; p ≤ 0.001) than the short birth depression group. Secondly, no evidence was found that the effects of hypothermia varied between the long birth depression and short birth depression group. Whole-body hypothermia did not improve brain injury or clinical outcome irrespective of the duration of birth depression. Finally, all babies without a heart rate at 5 min of resuscitation after birth either died or had severe disability.

Several adverse events during neonatal hospitalisation including persistent hypotension, pulmonary haemorrhage and persistent pulmonary hypertension were higher amongst babies who had long birth depression than those who had short birth depression. Amongst the babies who survived to have MR imaging, the brain injury was higher in the long depression group, except for cortical injury which was similar in both groups.

One explanation for this phenomenon is the concept of ‘Vulnerable baby in the womb’.^13^ It is possible that in LMICs, sub-optimal milieu, for example chronic intrauterine hypoxia, maternal undernutrition and low-grade inflammation contribute to fetal brain injury although our study was not designed to provide direct evidence for these. The encephalopathy due to the above factors adds on to the encephalopathy due to HIE or it has a multiplicative effect by potentiating the effect of asphyxia, and this is possibly the reason why outcomes were worse both in the long birth depression and the short duration depression groups. The differences between the long and short birth depression groups were at least partly due to the difference in the period of asphyxia. Thus, babies with moderate encephalopathy and short birth depression had lower death or disability at 18 months (31.9% versus 46.9%; p = 0.007) than those who had long birth depression, although the outcomes after severe encephalopathy were uniformly poor in both groups (84.6% versus 92.4%; p = 0.37).

Induced hypothermia was unable to reverse the changes due to birth asphyxia in both the long and the short duration groups either because secondary energy failure has either already occurred or due to the potentiating effects of nutritional and other inflammatory factors on the acute asphyxia-related injury. The high incidence of seizures at randomisation and absence of atrophic changes on MR imaging does suggest that the hypoxic injury is still intra-partum, although intra-partum sentinel events were documented only in small number of babies. Additionally, the average time of clinical seizure onset was much earlier in both depression groups than what is usually noted in HIE,^14^ suggesting occurrence of hypoxia might be earlier in labor rather than during birth process.

Observations of relatively normal cerebral energy metabolism at birth and subsequent neurological deterioration over the first few hours led to the discovery of the concept of secondary energy failure and development of induced hypothermia as a neuroprotective treatment for preventing such progression.^15^, ^16^, ^17^ Hence, LMIC clinicians at bed side observing progressive neurological deterioration in a baby who only had short birth depression may misinterpret this phenomenon as secondary energy failure and might initiate whole-body hypothermia to prevent this. As whole-body hypothermia was not neuroprotective it is likely that the deterioration may not be related to secondary energy failure. Further mechanistic research is required to understand this phenomenon.

In the HELIX trial all babies who had their first heartbeat after 5 min of birth either died or had severe disability. In contrast, 8 (35%) of the 23 babies without a heart rate at 10 min, survived without moderate or severe disability at 18 months in the NICHD NRN trial.^4^ Survival without disability has been reported in at least 20% of the babies without a heart rate at 10 min (n = 90) recruited to various high-income country trials.^18^^,^^19^ International Liaison Committee on Resuscitation recommends discontinuation of resuscitation only if these is no heart rate at 20 min after birth.^20^ The HELIX trial data suggest that further studies are required from LMICs on outcome of babies without a heart rate at 5 or 10 min to inform local policies.

There are some limitations of these data. Firstly, we did not have detailed intra-partum monitoring data as many infants were born outside the recruiting centre and were subsequently transferred to the participating centre. Hence only a small proportion of the babies had umbilical cord gases. Secondly, metabolic, and other genetic testing were not routinely performed in all babies, unless there was a strong clinical indication. Third, 18.8% from the short and 37.3% of babies from the long depression groups died before scans could be performed. Despite the survivor bias, babies in long depression group had more severe brain injury than the short depression group, which suggest that the difference between the groups were possibly more than what was detected in our study. Finally, we recruited only from public sector hospitals that cater to low-income populations, and these data may not be applicable to affluent population in LMICs.

There are several major health policy implications of these data, particularly as the HIE specific neonatal mortality remains unchanged (annual percentage change +0.1) in India between 2010 and 2019^21^ despite an overall reduction in neonatal mortality (annual percentage change of −32). Our hypothesis of ‘vulnerable baby in the womb’ may explain why HIE remains high in south Asia. It is possible that targeted intra-partum interventions and optimising the intra-partum care may prevent HIE in these settings. Secondly, poor outcome of babies without a detectable heartbeat at 5 min may inform resuscitation guidelines in LMICs.

Finally, current preclinical models of single acute hypoxic insult or infection-ischemia models may not reflect the clinical status in LMICs, instead models with multiple hypoxic insults in growth restricted models may be more appropriate. While such models do not exist as yet, Wassink et al. have reported a fetal lamb model of intermittent umbilical cord occlusion that leads to early onset of seizures, reduced recovery of EEG activity and brain injury.^22^ It is not known if hypothermia is neuroprotective in such models or indeed if intermittent hypoxia follows a different pathway of cerebral energy failure. The clinical heterogeneity of babies with HIE in LMICs is much higher than HICs.

In summary, long birth depression led to higher occurrence of severe encephalopathy, brain injury, mortality, and adverse neurodevelopmental outcomes than short birth depression in the HELIX trial. The outcome was not improved by whole-body hypothermia, irrespective of severity of birth depression.

## Contributors

CB analysed and interpreted the data and wrote the first draft. MM, SP, VK, RM, PMo and PMu assisted in interpretation of the data and preparation of the manuscript. SP assisted in the trial management. PB, CNK, SM, IJ, SCM, MS, RR, SS, RS supervised the site recruitments and assisted in interpretation the data. RC, SS assisted in neurodevelopmental outcome assessments and BPS and ARJ in magnetic resonance imaging. RRP was responsible for interpretation of the seizure data and PB was responsible for all statistical analysis. SSh and ST developed the trial protocol and supervised all aspects of the trial including data interpretation, neurodevelopmental outcomes, and preparation of the manuscript. All authors approved the final version of the manuscript. CB, SP, PMo, PB and ST accessed and verified the trial data. The corresponding author had full access to all the data in the study and had final responsibility for the decision to submit for publication.

## Data sharing statement

The trial protocol, statistical analysis plan, and case report forms are available from the corresponding author for a period of 10 years from the publication of this manuscript. Individual de-identified participant data (including data dictionaries) will be available from the corresponding author and will be shared with individuals and institutions with appropriate expertise for additional analysis, once sub-studies from the HELIX trial are reported.

## Declaration of interests

None.
