# Supplementary material for: Duration of birth depression and neurodevelopmental outcomes after whole-body hypothermia for hypoxic ischemic encephalopathy in India, Sri Lanka and Bangladesh – an exploratory analysis of the HELIX trial
Source: Lancet Reg Health Southeast Asia. 2023 Oct 4;20:100284. doi: 10.1016/j.lansea.2023.100284 (PMC10794099; doi:10.1016/j.lansea.2023.100284)
Supplement: Supplementary Table 1 [file mmc1.docx]

**Supplementary table 1. Baseline clinical characteristics, clinical and neurodevelopmental outcomes of babies with and without MRI performed within the long and short birth depression groups.**

|  | **Long birth depression group (n= 201)** | | | **Short birth depression group (n=207)** | | |
| --- | --- | --- | --- | --- | --- | --- |
|  | **MRI performed (n=116)** | **MRI not done (n=85)** | **P value** | **MRI performed (n=151)** | **MRI not done (n=56)** | **P value** |
| Maternal age, year | 25.0 ± 4.9 | 25.0 ± 4.5 | 0.970 | 23.8 ± 4.3 | 23.5 ± 4.5 | 0.686 |
| Booked pregnancies | 114 (98.3%) | 80 (94.1%) | 0.136 | 140 (92.7%) | 52 (92.9%) | 0.509 |
| Primigravida | 58 (50.0%) | 58 (68.2%) | 0.014 | 86 (57.0%) | 33 (58.9%) | 0.875 |
| Diabetes | 1 (0.9%) | 1 (1.2%) | 1.000 | 0 | 0 | - |
| Maternal pyrexia | 6 (5.2%) | 1 (1.2%) | 0.171 | 1 (0.7%) | 0 | 0.723 |
| Rupture of membranes >24h | 0 | 1 (1.2%) | 0.029 | 1 (0.7%) | 2 (3.6%) | 0.219 |
| Meconium-stained liquor | 32 (27.6%) | 37 (43.5%) | 0.019 | 30 (19.9%) | 11 (19.6%) | 0.990 |
| Reduced fetal movements | 8 (6.9%) | 9 (10.6%) | 0.619 | 6 (4.0%) | 1 (1.8%) | 0.498 |
| Funisitis | 16 (13.8%) | 11 (12.9%) | 0.046 | 31 (20.5%) | 8 (14.3%) | 0.206 |
| Perinatal sentinel events | 19 (16.4%) | 15 (17.6%) | 0.850 | 8 (5.3%) | 1 (1.8%) | 0.449 |
| Instrumental delivery | 14 (12.1%) | 15 (17.6%) | 0.089 | 9 (6.0%) | 2 (3.6%) | 0.733 |
| Caesarean delivery | 26 (22.4%) | 31 (36.5%) | 0.039 | 15 (9.9%) | 11 (19.6%) | 0.097 |
| **Infant size and condition** | | | | | | |
| Birth weight, grams | 2882 ± 439 | 2837 ± 529 | 0.513 | 2963 ± 422.0 | 2798 ± 428.9 | **0.013** |
| Gestational age, weeks | 38.6 ± 1.4 | 38.6 ± 1.4 | 0.715 | 38.8 ± 1.2 | 38.6 ± 1.1 | 0.359 |
| Head circumference, cm | 34.1 ± 1.5 | 34.6 ± 1.7 | **0.022** | 34.1 ± 1.4 | 34.5 ± 1.2 | 0.053 |
| Age at admission to the NICU, minutes | 110.5 ± 97.2 | 119.6 ± 98.7 | 0.517 | 173.4 ± 87.9 | 192.8 ± 76.7 | 0.146 |
| Cord pH | 7.0 ± 0.2 | 6.9 ± 0.2 | 0.082 | 7.1 ± 0.1 | - | - |
| Intubation at birth | 102 (87.9%) | 76 (89.4%) | 0.825 | 0 | 0 | - |
| Cardiac massage | 19 (16.4%) | 16 (18.8%) | 0.708 | 4 (2.6%) | 4 (7.1%) | 0.216 |
| Drugs during resuscitation | 25 (21.6%) | 18 (21.2%) | 1.000 | 5 (3.3%) | 1 (1.8%) | 1.000 |
| Males | 81 (69.8%) | 48 (56.5%) | 0.055 | 98 (64.9%) | 40 (71.4%) | 0.411 |
| Induced hypothermia | 51 (44.0%) | 49 (57.6%) | 0.064 | 71 (47.0%) | 31 (55.4%) | 0.348 |
| Severe encephalopathy | 23 (19.8%) | 43 (50.6%) | **<0.001** | 4 (2.6%) | 10 (17.9%) | <0.001 |
| Seizures at randomization | 81 (69.8%) | 54 (63.5%) | 0.365 | 128 (84.8%) | 36 (64.3%) | 0.002 |
| Age of seizure onset | 2 [1-5] | 3 [2-5] | 0.301 | 2 [1-3.5] | 4 [2-6] | <0.001 |
| **Clinical outcomes** | | | | | | |
| Death during hospitalization | 6 (5.2%) | 75 (88.2%) | <0.001 | 0 | 40 (71.4%) | <0.001 |
| Gastric bleeds | 10 (8.6%) | 42 (49.4%) | <0.001 | 18 (11.9%) | 26 (46.4%) | <0.001 |
| Persistent hypotension | 5 (4.3%) | 38 (44.7%) | <0.001 | 9 (6.0%) | 18 (32.1%) | <0.001 |
| Pulmonary haemorrhage | 5 (4.3%) | 38 (44.7%) | <0.001 | 7 (4.6%) | 20 (35.7%) | <0.001 |
| Persistent pulmonary hypertension | 8 (6.9%) | 21 (24.7%) | <0.001 | 4 (2.6%) | 7 (12.5%) | 0.010 |
| Severe thrombocytopenia | 9 (7.8%) | 17 (20.0%) | 0.018 | 11 (7.3%) | 11 (19.6%) | 0.020 |
| Persistent metabolic acidosis | 1 (0.9%) | 37 (43.5%) | <0.001 | 8 (5.3%) | 24 (42.9%) | <0.001 |
| **Outcomes at 18 months** | | | | | | |
| Death up until 18 months | 14 (12.1%) | 80 (94.1%) | <0.001 | 7 (4.6%) | 46 (82.1%) | <0.001 |
| Death or moderate or severe disability | 40 (34.5%) | 82 (96.5%) | <0.001 | 24 (15.9%) | 46 (82.1%) | <0.001 |
